# Pallidotomy for Dystonia: A Neglected Procedure?

**DOI:** 10.1002/mds.28409

**Published:** 2021-03-22

**Authors:** Marwan Hariz

**Affiliations:** ^1^ Department of Clinical Neuroscience Umeå University Umeå Sweden

Posteroventral pallidotomy in the treatment of Parkinson's disease, promoted in the prelevodopa era by Lars Leksell, in Lund, Sweden, in 1960,^1^ then forgotten after the introduction of levodopa therapy in the late 1960s, was rediscovered by Laitinen in Umea, Sweden, in 1985,^2^ thus marking the renaissance of surgery for PD in the postlevodopa era. Subsequent studies in nonhuman primates rendered parkinsonian with the neurotoxin 1‐methyl‐4‐phenyl‐1,2,3,6‐tetrahydropyridine, demonstrated the existence of segregated circuitry in the basal ganglia and confirmed a posteriori Leksell's and Laitinen's posteroventral pallidal target as being in the sensorimotor area of the globus pallidus internus.^3^, ^4^ Further global experience of the modern posteroventral pallidotomy in postlevodopa parkinsonian patients confirmed that its most striking effect was on the on–off fluctuations and the levodopa‐induced dystonia and dyskinesias.^5^ This was the very rational to implement posteroventral pallidotomy also for surgical treatment of dystonia in the modern era.^6^, ^7^, ^8^

The introduction of deep brain stimulation (DBS) of the ventral‐intermediate nucleus of the thalamus for parkinsonian and essential tremor in 1987,^9^ then DBS of the posteroventral pallidum in 1994^10^ and the subthalamic nucleus in 1995^11^ for advanced Parkinson's disease, and the subsequent global spread of DBS have overshadowed all previous ablative surgical procedures for movement disorders including posteroventral pallidotomy for dystonia. The latter was “replaced” eventually by DBS in the same target, introduced in 1999 by 3 neurosurgeons from 3 different centers, independently of each other.^12^, ^13^, ^14^ Since then, DBS has become the mainstream surgical procedure for virtually all types of dystonia, owing mainly to its safety profile compared with pallidotomy, especially when bilateral. Furthermore, a combination of, on the one hand, publications of a couple of randomized trials of DBS for dystonia providing “evidence‐based” results^15^, ^16^ and, on the other hand, relentless promotion of DBS by industry, leading to an almost complete loss of training in ablative surgery of younger neurosurgeons, has sealed the coffin over pallidotomy.^17^

Hence, the comprehensively documented review of Centen et al from Groningen, The Netherlands, on bilateral pallidotomy for dystonia published in this issue of the *Movement Disorders* journal comes as an eye‐opener about the potential of this procedure, not only as first choice when DBS is not available, but also in cases when DBS fails or has to be withdrawn because of infection and other hardware complications. An arrest of chronic DBS in dystonia patients can become a serious emergency.^18^ In fact, there are cases in which patients have developed fatal dystonic storm because the battery was depleted and a replacement battery was not readily available for financial or other reasons^19^ or because a severe status dystonicus failed to respond to DBS, resulting in 10% mortality.^20^ Bilateral pallidotomy — even staged — could then have saved the patients’ lives.

The systematic review of Centen et al truly finecombs the literature on bilateral pallidotomy for dystonia down to the smallest details. Ample details are provided in an extensive table in which the authors extracted from the literature all kinds of available clinical and epidemiological information on bilateral pallidotomy in dystonia patients, even on an individual level when available, including in patients with intractable status dystonicus who underwent bilateral pallidotomy, showing a 90% success rate in controlling the medication‐refractory dystonic storm. One is left with the perplexing impression about why such an efficacious procedure, even when staged, is so seldom used. One reason may be that the widespread use of DBS has resulted in a lack of training of younger‐generation neurosurgeons in the art of performing proper ablative procedures.

Two caveats in the review of Centen et al, acknowledged by the authors, are that, on the one hand, the published length of follow‐up of patients following pallidotomy has been rather short and, on the other hand, the rate of side effects after bilateral pallidotomy may have been downplayed in the reviewed literature. Be that as it may, one may still wonder what is worse in a patient who is literally dying from an intractable status dystonicus and DBS is not available^19^ or inefficient^20^: to undergo bilateral pallidotomy and suffer from dysarthria‐dysphonia complications or to succumb to the status dystonicus?

## Author Roles

Marwan Hariz: organization, execution; writing of the first draft; review and critique.

## Financial Disclosures (for the Preceding 12 Months)

The author has received travel expenses and honoraria from Boston Scientific for speaking at meetings.
